# Neuropsychological performance in young adults with cannabis use disorder

**DOI:** 10.1177/02698811211050548

**Published:** 2021-10-25

**Authors:** Ayla Kaya, Christelle Langley, Rebecca Crean, George Savulich, Francesca Cormack, Barbara J Sahakian, Barbara Mason

**Affiliations:** 1 Department of Psychiatry, University of Cambridge, Cambridge, UK; 2 The Pearson Center for Alcoholism and Addiction Research, Department of Molecular Medicine, The Scripps Research Institute, La Jolla, CA, USA; 3 Behavioural and Clinical Neuroscience Institute, University of Cambridge, Cambridge, UK; 4 Cambridge Cognition, Cambridge, UK

**Keywords:** Cognition, cannabis use disorder, CANTAB

## Abstract

**Background and Aims:**

Cannabis is a commonly used recreational drug in young adults. The worldwide prevalence in 18- to 25-year-olds is approximately 35%. Significant differences in cognitive performance have been reported previously for groups of cannabis users. However, the groups are often heterogeneous in terms of cannabis use. Here, we study daily cannabis users with a confirmed diagnosis of cannabis use disorder (CUD) to examine cognitive performance on measures of memory, executive function and risky decision-making.

**Methods:**

Forty young adult daily cannabis users with diagnosed CUD and 20 healthy controls matched for sex and premorbid intelligence quotient (IQ) were included. The neuropsychological battery implemented was designed to measure multiple modes of memory (visual, episodic and working memory), risky decision-making and other domains of executive function using subtests from the Cambridge Neuropsychological Test Automated Battery (CANTAB).

**Results:**

Our results showed that young adult daily cannabis users with CUD perform significantly poorer on tasks of visual and episodic memory compared with healthy controls. In addition, executive functioning was associated with the age of onset.

**Conclusions:**

Further research is required to determine whether worse performance in cognition results in cannabis use or is a consequence of cannabis use. Chronic heavy cannabis use during a critical period of brain development may have a particularly negative impact on cognition. Research into the persistence of cognitive differences and how they relate to functional outcomes such as academic/career performance is required.

## Introduction

Cannabis is one of the most commonly used drugs in young adults. The United Nations Office on Drugs and Crime (UNODC, 2020) reported that in 2018 approximately 192 million people worldwide aged between 15 and 64 years use non-medicinal cannabis. The prevalence of use was highest, approximately 35%, in young adults between the ages of 18 and 25 years, whereas it was just over 10% for adults age 26 (UNODC, 2020). Tetrahydrocannabinol (THC) is a cannabinoid receptor (CB1 and CB2) partial agonist, which is responsible for the psychoactive effects and dependency of cannabis (Sharma et al., 2012). Cannabis use has been linked to poor academic attainment, unemployment, legal trouble and increased risk of psychotic disorders (Hall and Degenhardt, 2009). Memedovich et al. (2018) conducted an overview of reviews investigating the harmful effects of cannabis. Indeed, 62 of the 68 included reviews identified harmful effects of cannabis use such as impaired driving, increased risk of stroke and some cancers, neural alterations, altered cognition and mental health problems. Hall et al. (2020) found that daily cannabis use during adolescence was related to poor cognition, which may affect educational achievement and occupation. Given the widespread use of cannabis among young adults, the impact of the harmful effects of cannabis use is particularly concerning.

It has been well established that cannabis use has detrimental effects on cognition. An early meta-analysis (Grant et al., 2003) comparing 623 cannabis users and 409 non-users demonstrated that cannabis users performed worse than non-users in a range of cognitive domains such as executive function, attention, learning and memory. A systematic review (Broyd et al., 2016) demonstrated consistent deficits in verbal memory across the literature, but more mixed results for working memory, decision-making and executive function. Scott et al. (2018) provide further evidence for the presence of poorer cognitive performance with cannabis use in a more recent meta-analysis examining a total of 2152 cannabis users and 6575 controls. Recently, Figueiredo et al. (2020) found small but significant correlations between chronic cannabis use and impairment in cognitive (but not motor) impulsivity, cognitive flexibility, attention, short-term memory and long-term memory. While meta-analyses are very useful to corroborate findings from the literature, they do have some limitations. The included studies often vary greatly on measures of length of abstinence, duration of cannabis use and frequency of cannabis use, which makes it difficult to compare the results across studies. Moreover, there is a large variety of cognitive domains and tests used in different studies. This heterogeneity often leads to inconsistent findings and reporting of small effect sizes. In addition, studies have shown that the age of onset may impact how cannabis use affects cognitive performance. Pope et al. (2003) demonstrated that early onset users (before 17) showed significantly decreased performance compared with late onset users after 28 days abstinence. However, once intelligence quotient (IQ) was controlled for, the differences ceased to be significant. This suggests a possible weak association between the age of onset and cognition. Gruber et al. (2012) showed decreased cognitive functioning in early onset users compared with late onset users; however, the early onset users also smoked greater quantities of cannabis compared with the late onset users, suggesting a potential confound. Fontes et al. (2011) compared early onset users (started smoking before age 15) and late onset users, with more comparable smoking habits, and showed decreased executive functioning in the early onset users.

However, many studies examine cannabis use, but do not require participants to have a specific, confirmed diagnosis of cannabis use disorder (CUD). CUD is characterised by the persistent desire to use the drug and disruption to daily activities such as work or education (American Psychiatric Association, 2013). It has been estimated that approximately 10% of cannabis users meet the *Diagnostic and Statistical Manual of Mental Disorders* (4th ed.; DSM-IV) criteria for CUD (Lopez-Quintero et al., 2011). Some previous work examining CUD has demonstrated poorer performance on cognitive tasks (Koenis et al., 2021), lower academic achievement (Hooper et al., 2014) and neural alterations (Ashtari et al., 2011; Koenis et al., 2021) in individuals with CUD. However, some of these studies have long periods of abstinence (6 months+) and may not be representative of the recent effects of chronic cannabis use. Indeed, Koenis et al. (2021) measure more recent effects; however, the average age of their sample is 40 and their study does not reflect the results from young adults, who have the highest prevalence of cannabis use (UNODC, 2020).

Importantly, in this study, we included only young adult daily users with a confirmed diagnosis of CUD to examine cognitive functioning in specific cognitive domains. Performance was compared with non-user controls, matched for premorbid IQ and sex, in a number of specific cognitive domains such as risky decision-making, multiple modes of memory and executive function using the well-validated Cambridge Neuropsychological Test Automated Battery (CANTAB) tests. Moreover, within the CUD group, we examined whether the age of onset of cannabis use was associated with cognitive performance. We hypothesise that the CUD group would perform worse on the neuropsychological battery compared with non-users. Moreover, we hypothesise that the age of onset would be associated with performance on measures of executive function.

## Methods

### Participants

Forty non-treatment seeking young adults with CUD and 20 healthy controls (Table 1) were recruited in San Diego, California, primarily via institutional review board (IRB)–approved newspaper advertisements and flyers. Participants between the ages of 21 and 30 were targeted. Groups were kept prospectively balanced for demographic variables such as age range, sex distribution, race, premorbid IQ, smoking status and family history of substance use disorder. Participants with CUD were included if they met the *Diagnostic and Statistical Manual of Mental Disorders* (5th ed.; *DSM*-5) criteria for CUD of moderate or greater severity (i.e. 4 or more symptoms), as assessed by a doctoral-level, trained clinician, typically used cannabis daily in past month and >200 times in past year. All study admissions were supervised by the Principal Investigator. Cannabis use was verified by a positive urine test at 50 ng/ml using the THC/Creatinine ratio. Controls were only included if they had used cannabis less than 5 times in their life and not within the last month. Participants were excluded if they had used other drugs (i.e. stimulants or hallucinogenics) more than 25 times in their life, assessed by the Illicit Drug Use Index (Clayton and Voss, 1981), smoked more than 10 cigarettes a day, assessed by the Fagerstrom Test for Nicotine Dependence (Fagerstrom, 2012), consumed more than 1 (female) or 2 (male) drinks per day or met the *DSM*-5 criteria for a major psychiatric disorder other than CUD. The study procedures were carried out in accordance with the Declaration of Helsinki and written informed consent was obtained from all participants. The ethics approval for this study was from the Scripps Research Institute–Institutional Review Board (TSRI-IRB), protocol number HSC#09-5229.

**Table 1.** Participant demographics.

		Cannabis (n = 39)	Controls (n = 20)	df	t-value/χ^2^	p-value
Sex	Males	26 (66.67%)	12 (60%)	1,59	0.26	0.61
	Females	13 (33.33%)	8 (40%)			
Age, years		22.77 (2.23)	24.25 (2.90)	30.85	2.00	0.05*
WAIS- Vocabulary		12.05 (2.36)	13.05 (3.02)	57	1.40	0.17
WRAT-Reading		105.23 (13.01)	111.20 (16.44)	31.7	1.41	0.17
Years of education		14.23 (1.40)	15.15 (2.06)	28.34	1.79	0.08
Alcoholic drinks/week		4.65 (4.56)	1.53 (2.15)	56.76	3.58	<0.001**
Smoking status	Yes	14	2	1,59	4.49	0.03*
	No	25	18			
Psychedelics	Yes	31	1	1,59	29.55	<0.001**
	No	8	19			
Opiates	Yes	10	1	1,59	3.71	0.05*
	No	29	19			
Stimulants	Yes	10	1	1,59	3.71	0.05*
	No	29	19			
Sedatives	Yes	4	0	1,59	2.20	0.14
	No	35	20			
Cocaine	Yes	26	2	1,59	17.91	<0.001**
	No	13	18			
Heroin	Yes	2	0	1,59	1.06	0.30
	No	37	20			
Employment status	Employed	16	9	2,59	2.50	0.29
	Unemployed	3	4			
	Student	20	7			
Age of onset of cannabis use		15.10 (2.44)				
Daily mean grams smoked in 90 days prior to study		1.02 (0.60)				
Years of daily cannabis smoked		4.68 (3.09)				

WAIS: Wechsler Adult Intelligence Scale; WRAT: Wide Range Achievement Test.

Mean (SD) or contingency tables are displayed. Drug use was recorded using the Illicit Drug Use Index (Clayton and Voss, 1981) and refers to lifetime drug use.

* p < 0.05; **p < 0.01.

### Power calculation

A power calculation with effect sizes in the range of 0.87 to 1.60 (based on previous work and personal communication with Dr. Susan Tapert, University of California – San Diego, CA) indicated that 9 to 24 participants are needed per group to detect similar effects. Thus, 60 young adults were recruited, 20 non-using controls and 40 with CUD, based on an intention to over-sample cannabis subjects to capture their potentially greater variability in performance due to varying duration and severity of cannabis use. This gives power of 0.80 to detect effect sizes with a two-sided α of .05, of d = .90 or greater for single-observation between-group comparisons.

### Cognitive assessment

All participants completed a battery of cognitive tasks. The neuropsychological battery implemented was designed to measure executive function, decision-making and memory using subtests from the touch screen–based CANTAB (Cambridge Cognition, 2012; Robbins et al., 1994, 1997; Sahakian and Owen, 1992). The test battery included the following tests: Delayed Matching to Sample (DMS), a 7-min task which assesses immediate and delayed visual memory; Paired Associates Learning (PAL), an 8-min task assessing visuospatial learning and episodic memory; Spatial Working Memory (SWM), a 4-min task assessing spatial working memory; Cambridge Gamble Task (CGT), an 18-min test assessing risky decision-making and Intra-Extra Dimensional Set Shift (IED), a 7-min test measuring cognitive flexibility. Full task descriptions are provided in the supplementary material. Illustrations of the CANTAB tasks are available at www.cambridgecognition.com.

### Procedure

Participants were pre-screened telephonically for preliminary eligibility and qualifying individuals were invited for a face-to-face screening visit. At the face-to-face screening, participants provided informed consent and were administered a structured clinical interview to assess the inclusion criteria for non-using controls or *DSM*-5 criteria for current CUD of moderate or greater severity (i.e. 4 or more symptoms), and to rule out significant psychiatric disorders that would warrant study exclusion. Participants IQ was also assessed using the WAIS (Wechsler Adult Intelligence Scale)-Vocabulary (Wechsler, 2008) and WRAT (Wide Range Achievement Test)-Reading (Wilkinson and Robertson, 2006). The number of years in education was also recorded. In addition, breath alcohol concentration and urine drug screens were obtained to validate self-report of substance use. On the day of testing, 1 week after the screening visit, participants would attend the study visit at the laboratory. A study physician would conduct a similar screening to clear the participant for study participation. The participant then provided a urine sample for THC/Cr determination. Participants were also asked to complete the Timeline Follow-back Interview (TLFB) (Fals-Stewart et al., 2000; Sobell and Sobell, 1992), which measures usage patterns 90 days prior to the study visit. The screening would last approximately 45 min after which the participant would begin completing the neuropsychological tests. The testing session lasted approximately 2 h 15 min. The tasks were presented in the following fixed order: CGT, IED, SWM, PAL and DMS. Participants were remunerated for their time.

### Statistical analysis

The study design was a parallel group design. Data analyses were conducted in SPSS software, version 24. One cannabis user was excluded from the analysis due to smoking cannabis on the day on CANTAB testing; therefore, the final sample consisted of 39 CUD participants and 20 healthy controls. Demographic data (Table 1) were compared between groups using t-tests for age, IQ and alcohol use, and Fisher’s exact test for sex, lifetime drug use and smoking status. For group comparisons, the number of stages completed on the IED task was analysed using Fisher’s exact test. The remaining 11 outcome variables, for group differences, were tested using univariate analysis of covariance (ANCOVA), with group (cannabis or control) as the independent variable and each outcome measure as the dependent variable while controlling for age, lifetime drug use, smoking status and alcohol use (drinks per week), due to the significant differences between groups. Lifetime drug use was assessed by the Illicit Drug Use Index (Clayton and Voss, 1981) and smoking status was assessed by the Fagerstrom Test for Nicotine Dependence (Fagerstrom, 2012). Due to the number of tests administered for the group comparisons, the Benjamini–Hochberg (Benjamin and Hochberg, 1995) procedure was applied and the false discovery rate (FDR) set a priori at q < .05. All reported significant differences survived the FDR correction and effect sizes are reported as partial eta squared ( $\eta_{p}^{2}$ ). To determine whether a relationship between the age of onset and cognition existed, a partial correlation controlling for age, lifetime drug use, smoking status and alcohol use was conducted. A separate Benjamini–Hochberg FDR correction with q < .05 was conducted for the correlations.

## Results

Demographically, participants were matched for IQ, prorated from the WAIS-Vocabulary and WRAT-Reading, and sex, but there was a significant difference in age between the CUD and non-user control groups, which was therefore used as a control variable in the analyses (Table 1). In addition, there were group differences between lifetime drug use, smoking status and alcohol use and as such were included as covariates. Shapiro-Wilk’s and Levene’s tests for homogeneity were non-significant, indicating the assumptions for analysis of variance (ANOVA) were not violated.

## Visual memory (CANTAB DMS)

**Figure 1.**
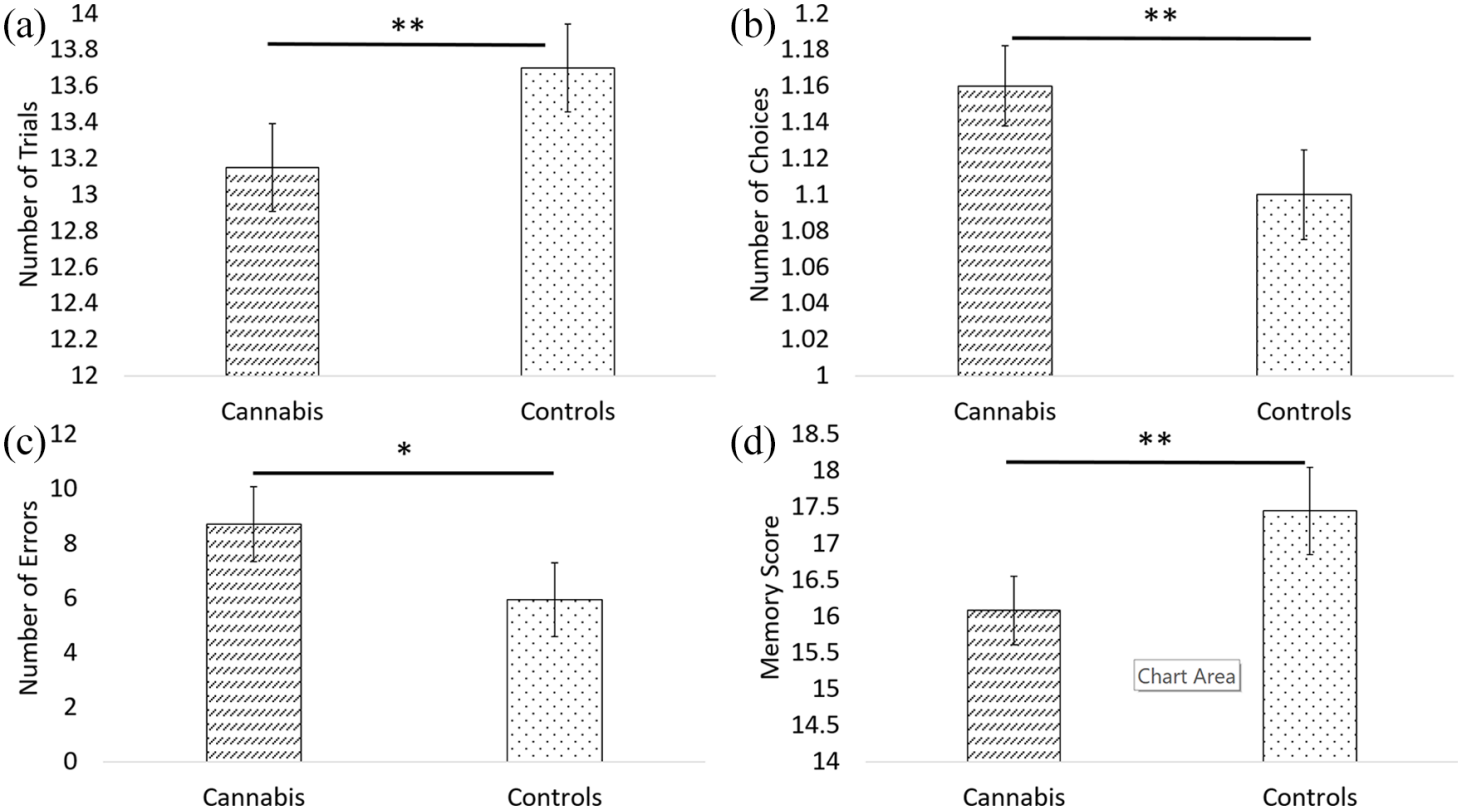
Performance on the CANTAB DMS and CANTAB PAL tasks. (a) DMS Total correct; (b) DMS mean choices to correct; (c) PAL total errors adjusted; (d) PAL first trial memory score (higher score better). Cannabis: n = 39; controls: n = 20. *p < 0.05; **p < 0.01.

The analysis of performance on the CANTAB DMS (Figure 1) demonstrated that the cannabis-dependent group had significantly poorer memory performance compared with the control group for both the total correct outcome measure (F(1, 48) = 21.57, p < 0.001, $\eta_{p}^{2}$ = .31) and the mean choices to correct outcome measure (F(1, 48) = 14.72, p < 0.001, $\eta_{p}^{2}$ = .24). There was no relationship between the age of onset for the total correct trials (R = 0.34, p = 0.07) and the mean choices to correct outcome variable (R = −0.35, p = 0.06).

## Episodic memory (CANTAB PAL)

The results from the ANCOVA (performance displayed in Figure 1) showed significantly lower scores in the cannabis-dependent group compared with controls for the PAL total errors adjusted (F(1, 48) = 6.03, p = 0.02, $\eta_{p}^{2}$ = .11) and first trial memory score (F(1, 48) = 6.67, p = 0.01, $\eta_{p}^{2}$ = .12). Moreover, there was no relationship between the age of onset for total errors adjusted (R = −0.10, p = 0.62) or first trial memory score (R = 0.06, p = 0.74).

## Working memory (CANTAB SWM)

Similar to performance on the PAL and DMS results (Figure 1), the cannabis-dependent group showed significantly worse memory performance compared with controls on the SWM task (Table 2) (between search errors: F(1,48) = 4.59, p = 0.04, $\eta_{p}^{2}$ = .09). There were no statistically significant differences between the two groups on the strategy score (F(1,48) = 2.00, p = 0.16, $\eta_{p}^{2}$ = .04). There was a significant negative correlation between strategy score and the age of onset (R = −0.46, p = 0.01). The negative correlation between the age of onset and between search errors (R = −0.46, p = 0.01) did not survive the FDR correction at q = .05.

**Table 2.** Performance on CANTAB SWM, CANTAB CGT and CANTAB IED.

		Cannabis (n = 39)	Control (n = 20)
CANTAB SWM	Between Search Errors	21.49 (15.99)	13.70 (13.67)
	Strategy Score	31.36 (5.47)	29.15 (6.12)
CANTAB CGT	Overall Proportion Bet	0.74 (0.17)	0.62 (0.16)
	Deliberation Time	1763.49 (713.14)	1487.56 (540.78)
	Quality of Decision Making	0.94 (0.08)	0.95 (0.08)
CANTAB IED	Pre-ED Errors	7.71 (3.86)	6.10 (2.15)
	ED Errors	6.31 (8.63)	7.45 (9.10)
	Failed Stage 8	5	3

CANTAB: Cambridge Neuropsychological Test Automated Battery; SWM: Spatial Working Memory; CGT: Cambridge Gamble Task; IED: Intra-Extra Dimensional Set Shift; FDR: false discovery rate.

Mean (SD) or contingency tables are displayed. CGT deliberation time p = 0.03 and SWM between search errors p = 0.04 did not survive FDR at q < .05.

## Risky decision-making (CANTAB CGT)

The cannabis-dependent group showed some increased deliberation time (F(1, 48) = 4.80, p = 0.03, $\eta_{p}^{2}$ = .09) compared with controls; however, this did not survive FDR correction at q = .05. There were no significant differences between groups on the overall proportional bet (F(1, 48) = 0.86, p = 0.36, $\eta_{p}^{2}$ = .02) and the quality of decision-making (F(1, 48) = 0.62, p = 0.44, $\eta_{p}^{2}$ = .01) (Table 2). We observed a significant positive correlation between the age of onset and quality of decision-making (R = 0.45, p = 0.01). The negative correlation between deliberation time and the age of onset (R = −0.40, p = 0.03) did not survive FDR correction at q = .05. There was no significant correlation between the age of onset and overall proportion bet (R = 0.07, p = 0.70).

## Cognitive flexibility (CANTAB IED)

There were no significant differences between the cannabis-dependent and control group on the number of errors made (pre-ED errors: F(1, 48) = 0.55, p = 0.46, $\eta_{p}^{2}$ = .01; EDS errors: F(1, 48) = 0.20, p = 0.66, $\eta_{p}^{2}$ < .01). Furthermore, there were no statistically significant differences in the number of participants passing the ED shift at stage 8 (χ^2^(2, N = 59) = .05, p = 0.82) (Table 2). The marginal correlation between ED shift errors and the age of onset (R = −0.36, p = 0.05) did not survive FDR correction and there was no significant correlation between pre-ED errors and the age of onset (R = −0.35, p = 0.06).

## Discussion

This study examined cognition in young adult daily users with CUD and non-user control participants in a number of specific cognitive domains such as risk-taking, memory and executive function using the well-validated CANTAB battery. We also examined the relationship between the age of onset and cognition. The results of the group comparisons showed significantly poorer performance in the cannabis group in visual and episodic memory, whereas there were no performance differences in executive functioning. The results demonstrated a clear pattern of poorer memory performance in the cannabis group compared with the controls across a number of tests. For the cannabis group compared with controls in the DMS, a test of visual memory, we observed significantly reduced task accuracy. The PAL, a test of episodic memory, showed that participants with CUD completed fewer trials on the first attempt, as well as making increased errors across the task. Our results demonstrated no significant differences between the CUD group and controls in executive function as measured by the IED (EDS errors), SWM (strategy score) and the quality of decision-making component of the CGT, thereby suggesting that multiple domains of memory are particularly affected by chronic cannabis use, whereas executive function remains largely intact. Indeed, there are multiple reviews in the literature that have shown consistent detrimental effects for memory domains, but more mixed results for executive functioning (Broyd et al., 2016; Curran et al., 2016), as several reviews suggest that executive function is indeed affected by cannabis use (Figueiredo et al., 2020; Grant et al., 2003; Scott et al., 2018). However, it is important to note that many of these reviews focus on residual cognitive effects following a period of prolonged abstinence and are therefore not directly comparable with the present study. Broyd et al. (2016) concluded from their review that cognitive impairments may persist for approximately a week with chronic cannabis use, but are often resolved with longer periods of abstinence. However, it is unclear whether the studies reviewed used participants diagnosed with CUD.

Moreover, cognitive performance is associated with frequency, quantity and duration of use, as well as the age of onset (Thames et al., 2014; Wagner et al., 2010). As such, the heterogeneity across studies on measures such as of length of abstinence, duration of cannabis use, frequency of cannabis use and age on onset makes it very difficult to compare the results across studies. Indeed, Scott et al. (2018) state that there is a need for standardised cannabis use metrics or innovative techniques to measure cannabinoid levels to better characterise cannabis use across studies. Similarly, Loflin et al. (2020) advocate for more standardised outcome measures of CUD clinical trials. More standardised measures of cannabis use across studies may better elucidate the relationship between cognitive function and cannabis use. In an attempt to achieve this, this study only included participants with a confirmed diagnosis of CUD, assessed by the *DSM*-5. Only a few studies have examined CUD specifically and show poorer performance on cognitive tasks (Koenis et al., 2021), lower academic achievement (Hooper et al., 2014) and neural alterations (Ashtari et al., 2011; Koenis et al., 2021) in individuals with CUD. However, the results are also not directly comparable as two use long periods of abstinence (6 months+, Ashtari et al., 2011; Hooper et al., 2014) and one has an older sample (mean age: 40, Koenis et al., 2021).

There is a slightly broader pattern of poorer performance that did not survive the multiple comparisons at q < .05, but does survive at q < .10 (Genovese et al., 2002). Specifically, the CUD group had increased deliberation time on the CANTAB CGT and increased between search errors on the CANTAB SWM task. This may suggest that is poorer performance in more widespread cognitive domains that may affect young adult daily users with CUD. A previous study using the CANTAB SWM task in cannabis users reported no statistically significant differences between controls and cannabis users; however in their study, most participants did not fulfil the criteria for CUD (Grant et al., 2012). Harvey et al. (2007) found significantly poorer performance in adolescent cannabis users compared with controls, perhaps suggesting that the neurodevelopmental period is important. Similarly, Becker et al. (2014) found reduced spatial working memory performance in young adult daily cannabis users. Grant et al. (2012) also employed the CANTAB CGT and, similar to our results, showed no difference in the proportion bet between cannabis users and non-users, but they did not report on the deliberation time.

The participants were asked to refrain from using cannabis on the day of testing and this was confirmed this with the TLFB interview. Nonetheless, there was no objective way to confirm that participants were compliant, given the long half-life of THC and its metabolites in urine. However, our participants typically function under the influence of chronic heavy cannabis use, and from this perspective, our results represent this sample’s baseline performance on the tasks administered.

The results from the correlation analyses demonstrated a significant association between the age of onset and executive functioning on the SWM (strategy score) and the CGT (quality of decision-making). There was a weak correlation between ED errors and the age of onset, which did not survive correction for multiple comparisons, but which further supports a possible association between executive functioning and the age of onset. It is possible that cannabis users have an executive vulnerability that may predispose them to cannabis use. The few studies that have examined cognition between early and late onset users have demonstrated decreased executive functioning in the early onset group compared with the late onset group (Fontes et al., 2011; Gruber et al., 2012; Pope et al., 2003). However, these studies do not address whether the decreased executive function is due to the cannabis use or due to a specific behavioural phenotype being more susceptible to early cannabis use. Fontes et al. (2011) showed decreased executive function in early onset users compared with late onset users, despite similar levels of cannabis use, suggesting that the differences in executive function are more related to the age of onset than cannabis use. A study of stimulant abuse has indicated that increased behavioural impulsivity may predispose individuals to stimulant use (Ersche et al., 2010), thereby suggesting that a specific behavioural phenotype may result in drug use rather than being caused by drug use. However, this study did not employ a longitudinal design that would best be able to explore whether poorer cognitive performance predisposes individuals to cannabis use. Indeed, Becker et al. (2018) did employ a longitudinal design and examined 26 heavy cannabis users and 31 age- and sex-matched controls. The results showed poorer cognitive performance at both baseline and follow-up in the cannabis users; however, this cognitive decline was not associated with cannabis use itself. As such the authors argue that poorer cognitive performance represents enduring vulnerabilities, especially among chronic heavy users. Importantly, earlier age of onset of cannabis use was associated with poorer performance in the domains of verbal learning and memory. The findings from a meta-analysis of longitudinal studies (Castellanos-Ryan et al., 2017) suggest that the link between cannabis use and cognition can be bidirectional; cognitive vulnerabilities may lead to earlier age of onset, which in turn can negatively impact cognitive functioning in later life.

Despite the heterogeneity in the literature, there are some obvious detrimental effects on cognition for chronic cannabis use, particularly with early onset. Human brains are still in development into adolescence and early adulthood, for example, the frontal lobes are structures that develop late (Shaw et al., 2008). Therefore, the effects of cannabis on cognition are particularly pertinent in adolescents and young adults given this critical neurodevelopmental period (Lubman et al., 2015). It is possible that cannabis use and specifically CUD during such a critical neurodevelopment period may mean that poor cognitive functioning has a particularly devastating effect. However, research into the persistence of these cognitive differences and how they relate to functional outcomes such as academic and career performance is required.

## Conclusion

Importantly, this study used a sample of young adult daily users with a confirmed diagnosis of CUD and assessed cognition with well-validated neuropsychological tests. The results showed worse cognitive performance in visual and episodic memory in the CUD group compared with the control group. Executive functioning was related to the age of onset, but further research is required to determine whether poorer cognitive functioning results in cannabis use or is a consequence of cannabis use. Moreover, research into the persistence of these cognitive differences and how they relate to functional outcomes such as academic and career performance is required.

## Supplemental Material

sj-docx-1-jop-10.1177_02698811211050548 – Supplemental material for Neuropsychological performance in young adults with cannabis use disorder Supplemental material, sj-docx-1-jop-10.1177_02698811211050548 for Neuropsychological performance in young adults with cannabis use disorder by Ayla Kaya, Christelle Langley, Rebecca Crean, George Savulich, Francesca Cormack, Barbara J Sahakian and Barbara Mason in Journal of Psychopharmacology
