# Supplementary material for: Neuropsychological performance in young adults with cannabis use disorder
Source: J Psychopharmacol. 2021 Oct 25;35(11):1349–55. doi: 10.1177/02698811211050548 (PMC8600580; doi:10.1177/02698811211050548)
Supplement: sj-docx-1-jop-10.1177_02698811211050548 – Supplemental material for Neuropsychological performance in young adults with cannabis use disorder [file sj-docx-1-jop-10.1177_02698811211050548.docx]

Neuropsychological performance in young adults with cannabis use disorder.

Supplementary Material:

**CANTAB Delayed Matching to Sample (DMS)**

The CANTAB DMS is a seven-minute task which assesses immediate and delayed visual memory. It tests both the ability to perform simultaneous visual matching and short-term recognition for non-verbal patterns. Participants are shown an abstract visual pattern (sample) followed by four similar patterns. The participant must select the pattern which exactly matches the sample. In some trials, the sample and the choice patterns are shown simultaneously, in others there is a delay of 4 or 12 seconds before the four choices appear before the participant. There are 4 practice trials followed by 20 counterbalanced test trials, including 5 simultaneous and 5 at each of the 3 delay intervals (0, 4, 12 second delays). The outcome measures chosen were total number correct (the total number of times a subject chose the correct answer on their first box choice, calculated across all assessed trials simultaneous presentation and all delays) and mean number of choices to correct (the mean number of choices that the subject made on each trial, including the correct choice, calculated across all trials where the subject eventually made the correct choice simultaneous and all delays).

**CANTAB Paired Associated Learning (PAL)**

The CANTAB PAL is an eight-minute task assessing visuospatial learning and episodic memory. Boxes are displayed on the screen and opened in a random order. One or more boxes will contain a pattern. The patterns are subsequently displayed in the middle of the screen and the participant must select the box in which the pattern was previously presented. If the participant makes an error, the boxes are opened in the same order again. This is to remind the participant of the locations of the patterns. There is a 2 pattern practice and then 2, 4, 8 and 8 patterns assessed, participants get 4 attempts at each level before the task is ended. The outcome measures chosen were total errors adjusted (the number of times the subject chose the incorrect box for a stimulus on assessment problems, plus an adjustment for the estimated number of errors they would have made on any problems, attempts and recalls they did not reach) and the first trial memory score (the number of times a subject chose the correct box on their first attempt when recalling the pattern locations, calculated across all assessed trials). This test has been extensively validated and is a good indicator of mild cognitive impairment and Alzeheimer’s disease (Barnett et al., 2016).

**CANTAB Spatial Working Memory (SWM)**

The CANTAB SWM is a four-minute task assessing spatial working memory. Task performance requires the retention and manipulation of visuospatial information. Coloured boxes are shown on screen and participants must select a box with a token. The colour and position of the boxes used are changed from trial to trial to discourage the use of stereotyped search strategies. There are two 3 box practice trials and then a 4, 6 and 8 box assessed trial. The outcome measures chosen were between search errors (the number of times the subject incorrectly revisits a box in which a token has previously been found, calculated across all assessed trials) and strategy score (the number of times a subject begins a new search pattern from the same box they started with previously. If they always begin a search from the same starting point, we infer that the subject is employing a planned strategy for finding the tokens. Therefore, a low score indicates high strategy use (1 = they always begin the search from the same box), a high score indicates that they are beginning their searches from many different boxes. Calculated across assessed trials with 6 tokens or more).

**CANTAB Cambridge Gamble Task (CGT)**

The CGT is an 18 minute test where participants are presented with ten boxes, some red and some blue. The ratio of red and blue boxes varies between stages however, one box contains a yellow token. Participants must use the 'Red' and 'Blue' buttons at the bottom of the screen to choose the box colour in which they think the token is hidden. Participants begin with 100 points to bet on their decision. Points will either be added or taken away to their total score, depending on their decision and where the token is actually hidden. In the CGT the odds are displayed explicitly, as such there is no working memory requirement to remember the odds ratio. There are 8 practice trials (4 decision-only and 4 ascending bet practice trials) and 36 assessed ascending bet trials followed by 4 descending bet practice trials and 36 assessed descending bet trials. Outcome measures chosen were quality of decision-making (the proportion of all trials where the subject chose the majority box colour, and is calculated over all assessed trials from both the ascending and descending conditions); overall proportional bet (the mean proportion of current points gambled by the subject and is calculated over all assessed trials from both the ascending and descending conditions) and deliberation time (the median latency from presentation of the boxes to the subject’s selection of the colour on which to bet calculated over all assessed trials in both the ascending and descending conditions). The CGT has been shown to be sensitive to impairment in substance use disorder (Rogers et al., 1999) and gambling addiction (Lawrence et al., 2009).

**CANTAB Intra/Extra Dimensional Set Shift (IED)**

The IED is a seven-minute test measuring cognitive flexibility and represents a computerised version of the Wisconsin Card Sorting test (Berg, 1948). It features rule acquisition and reversal where visual discrimination and attentional formation is required. Participants are presented with two artificial dimensions including pink shapes and white lines. Through trial and error the participant must select the correct rule. After six correct responses, the stimuli and/or rule changes. There are 9 stages to the task. Initially the task presents simple stimuli with just one dimension (pink shapes). These later change to compound stimuli (white lines overlaid on pink shapes). Early in the task the shifts are intra-dimensional (ID) (pink shapes are relevant) and then at stage 8 a crucial extra-dimensional (ED) shift (white lines become relevant) occurs. During each stage the learning criterion is 6 consecutive correct answers, there are a total of 50 trials per stage. If the participant has not reached the learning criterion within the total trials the participant fails that stage and the task is stopped. Adjusted errors are assigned to the subsequent stages. Outcomes measures chosen were the number of pre-ED errors (all errors stage 1-7), ED shift errors (errors at stage 8) and failures at stage 8. The IED is a well-validated measure of set-shifting in both humans and animals (Aoki et al., 2015; Dias et al., 1996; Hampshire and Owen, 2006).

References:

Aoki, S., Liu, A. W., Zucca, A., Zucca, S., & Wickens, J. R. (2015). Role of striatal cholinergic interneurons in set-shifting in the rat. Journal of Neuroscience, 35:9424–9431.

Barnett, J. H., Blackwell, A. D., Sahakian, B. J., & Robbins, T. W. (2016). The paired associates learning (PAL) test: 30 years of CANTAB translational neuroscience from laboratory to bedside in dementia research. In Current Topics in Behavioral Neurosciences: 449–474.

Berg, E. A. (1948). A simple objective technique for measuring flexibility in thinking. *Journal of General Psychology*, *39*: 15–22.

Dias, R., Robbins, T. W., & Roberts, A. C. (1996). Dissociation in prefrontal cortex of affective and attentional shifts. Nature. 380:69-72.

Hampshire, A., & Owen, A. M. (2006). Fractionating attentional control using event-related fMRI. Cerebral Cortex, 16:1679–1689.

Lawrence, A. J., Luty, J., Bogdan, N. A., Sahakian, B. J., & Clark, L. (2009). Problem gamblers share deficits in impulsive decision-making with alcohol-dependent individuals. Addiction, 104: 1006–1015.

Rogers, R. D., Everitt, B. J., Baldacchino, A., Blackshaw, A. J., Swainson, R., Wynne, K., Baker, N. B., Hunter, J., Carthy, T., Booker, E., London, M., Deakin, J. F. W., Sahakian, B. J., & Robbins, T. W. (1999). Dissociable deficits in the decision-making cognition of chronic amphetamine abusers, opiate abusers, patients with focal damage to prefrontal cortex, and tryptophan-depleted normal volunteer Evidence for monoaminergic mechanisms. Neuropsychopharmacology, 20: 322–339.
